# B Cell Dysfunction Associated With Aging and Autoimmune Diseases

**DOI:** 10.3389/fimmu.2019.00318

**Published:** 2019-02-27

**Authors:** Shiliang Ma, Chengwei Wang, Xinru Mao, Yi Hao

**Affiliations:** Department of Pathogen Biology, School of Basic Medicine, Tongji Medical College, Huazhong University of Science and Technology, Wuhan, China

**Keywords:** aging, autoimmunity, BCR repertoires, T-bet, B cells

## Abstract

Impaired humoral responses, as well as an increased propensity for autoimmunity, play an important role in the development of immune system dysfunction associated with aging. Accumulation of a subset of atypical B cells, termed age-associated B cells (ABCs), is one of the key age-related changes in B cell compartments. ABCs are characterized by their distinct phenotypes, gene expression profiles, special survival requirements, variations in B cell receptor repertoires, and unique functions. Here, we summarize recent progress in the knowledge base related to the features of ABCs, their potential role in immune senescence, and their relationship with autoimmune diseases.

## Introduction

Humoral immune responses mediated by B cells are important for adaptive immunity. B cells produce a diverse set of antibodies, which help in effectively eliminating antigens including pathogens. In addition, B cells play an indispensable role in the immune system via presentation of antigens and secretion of cytokines (1–3). Aging is a complex process accompanied by a functional decline in multiple physiological systems. In aged individuals, a spectrum of immune system alterations, termed “immune senescence,” result in a blunted adaptive immune response, an increased tendency for inflammatory responses, enhanced susceptibility to infections, and an increased production of autoantibodies (4–7). Multiple factors may contribute to these immune activity changes. T cells have been shown to participate in immune senescence. However, the role of B cells in this respect remains unclear. Recent findings illustrate conspicuous shifts in B cell subsets in the elderly, suggesting that age-related changes in B cells may contribute to immune senescence (8–10). The discovery of a subset of B cells, termed age-associated B cells (ABCs), has drawn significant attention in recent years. Initially isolated from aged donors and found to be closely associated with immune senescence, these cells were expected to provide a novel therapeutic avenue for autoimmune diseases. With due consideration to various aspects of their potential, we review previous findings and provide an insight into the properties, functions, and related signaling pathways, which may assist in developing a better understanding of this subset of B cells (Table 1).

**Table 1:** Properties of ABCs in aging and in autoimmune diseases.

	**In aging**	**In autoimmune diseases**
Described in	Mice (4, 9–12)	Mice (10) and patients (13–17)
Phenotype	CD21^−^/35^−^CD23^−^ (4, 9, 11) and CD11c^+^ CD21^−^ T-bet^+^ (10, 12)	CD11c^+^CD11b^+^CD21^low^T-bet^+^ (10, 13), CXCR5^−^CD21^−^CD11c^+^ (14), and CD11c^+^FcRL4^+^ (17)
BLyS receptor	BR3 and TACI (9)	BR3^high^, TACI^int^, and BCMA^low^ (13)
Production of autoantibody	Yes (10)	Yes (10, 13, 14)
Secretion of cytokines	TNF-α, IL-4, IL-10 (4, 9)	N/A
Presenting antigens	Yes (9, 12)	N/A
Response to TLR stimulation	Yes, TLR9, and TLR7 (9, 10)	Yes, TLR7 (10, 14)
Response to BCR stimulation	Poor (9)	N/A

## Changes in B Cell Compartments During Aging

### Impaired B Cell Development in the Bone Marrow With Aging

Normally, B2 B cell development occurs in a fixed order. Emerging from lymphoid-biased hematopoietic stem cells (HSC), these newly generated cells successively develop into pro-B cells, pre-B cells, and immature B cells in the bone marrow, followed by the exit of immature B cells from the bone marrow and completion of maturation. The mature B cells are composed of two peripheral pools: marginal zone B cells and follicular B cells (2). Different subsets of B cells have unique functions in the human body and contribute to the balance and efficacy of immune response.

**Figure 1:**
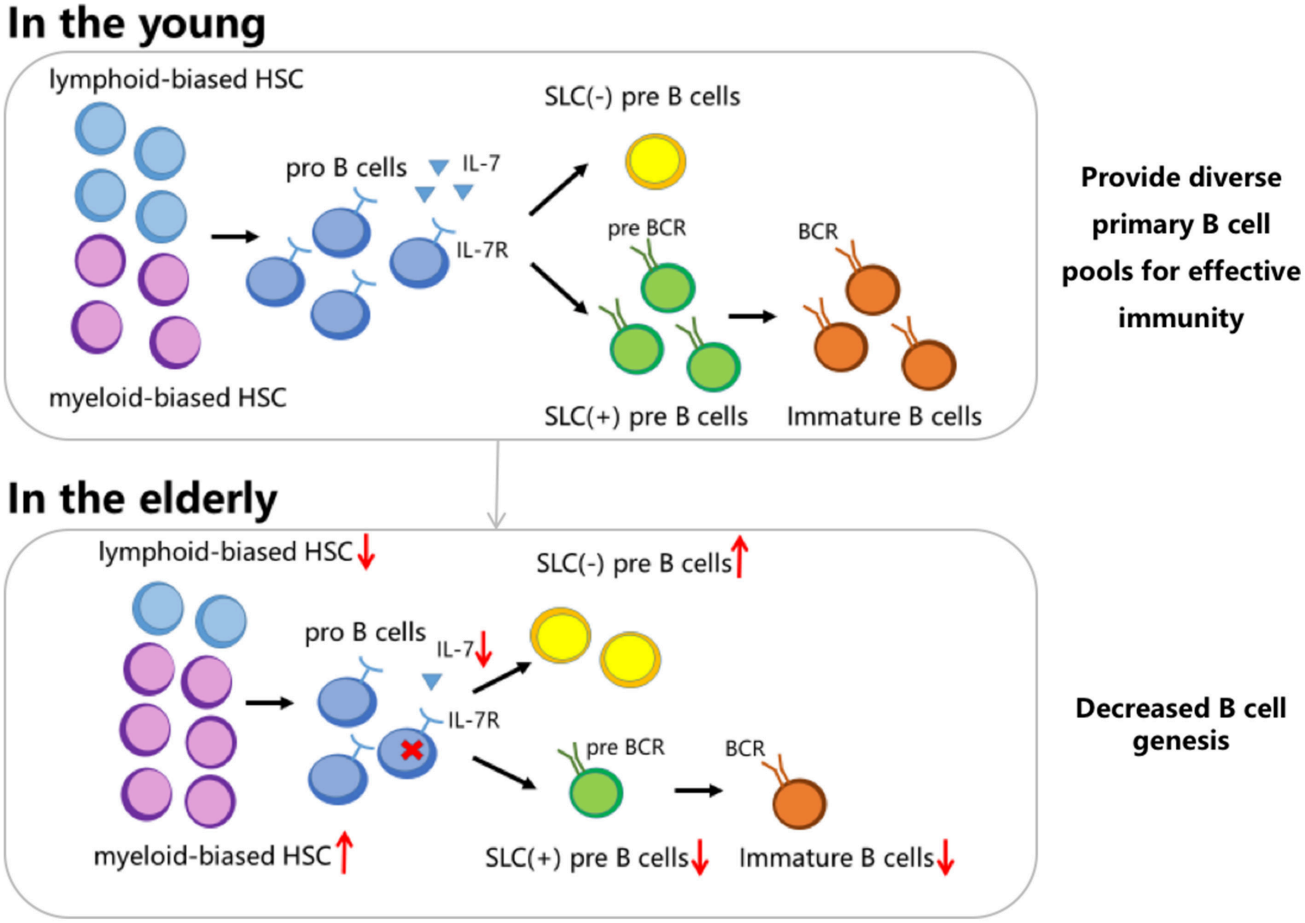
Altered renewal rate of B cells in the bone marrow of the elderly. The phenomenon can be interpreted in three ways. Firstly, HSC switch from lymphoid-biased to myeloid-biased with aging. Secondly, the ability of aged pro-B cells to respond to IL-7 is impaired, and the release of IL-7 from stromal cells in the bone marrow is decreased. Thirdly, there is a deficit of SLC^+^ precursor B cells and an accumulation of SLC^−^ cells.

Although total peripheral B cell counts remain relatively stable in adulthood (8), aging has been associated with a decline in B cell production in the bone marrow (18). This phenomenon is ascribed to both cell-intrinsic changes and alterations in lymphoid organ microenvironments, which may be interpreted in 3 different ways (19, 20). Firstly, analysis of clonal composition of hematopoietic stem cells (HSCs) from aged mice showed that age-associated switching of HSCs from lymphoid-biased cells to myeloid-biased ones, reduced the source of B cell production (21), which was partly due to *PAX5* expression being significantly attenuated in older individuals (22). Secondly, *in vitro* studies indicated that the ability of pro-B cells to respond to IL-7 was impaired (23) and that the release of IL-7 from stromal cells in the bone marrow was decreased due to aging (24). These factors reduce pro-B cell proliferation in the elderly. Thirdly, lower renewal rates and immune efficacy of B lymphocytes are responsible for a decrease in surrogate light chain (SLC)^+^ precursor B cells and an accumulation of SLC^−^ B cells. Two pathways associated with the impaired balance between SLC^+^ pre-B cells and SLC^−^ cells have been corroborated to prove this hypothesis: (1) Inhibitor of DNA binding 2 (ID2) in precursor B cells increases with age and blocks the activity of E2A, an essential transcription factor regulating the transcription of SLC genes, λ5 and VpreB (25–27). Diminution of SLC causes the loss of pre-B cell receptors, limiting the expansion and further development of pre-B cells, and reducing the generation of B cells with normal functions (25). (2) Increased secretion of TNF-α by old follicular B cells (28) induces apoptosis of SLC^+^ pro-B cells in the bone marrow (4), followed by the accumulation of SLC^−^ B cells that impede the production of immature B cells (29). The signaling pathways mentioned above indicate that age-related changes in the bone marrow, leading to impaired development, and function of B cells, may facilitate the process of immune senescence (Figure 1).

### Accumulation of ABCs in the Periphery During Physiological Aging

Hao et al. and Rubtsov et al. reported that a novel subset of B cells, termed age-associated B cells (ABCs), accumulated in aged mice (9, 10). These B cells first accumulated in the spleen and increased significantly in the bone marrow with age (4, 9). ABC phenotypes are distinct from other B cell subsets. Hao et al. defined CD43^−^CD21^−^/35^−^CD23^−^ B cells as ABCs (9), while Rubtsov et al. described them as CD11b^+^CD11c^+^ B cells (10). These 2 groups found that ABCs expressed similar levels of IgM and lower levels of IgD compared to follicular B cells (9, 10). In addition, cell cycle analyses showed that ABCs were quiescent, suggesting that they are not a subset of self-renewing cells (9). Because ABCs were explored using mouse models, the existence of similar cells in aged humans may need confirmation.

More interestingly, B cells with phenotypes similar to that of ABCs appear in both mice and humans, during the course of certain autoimmune diseases (10, 13, 14), and following some viral infections (30, 31). In this review, we focus on ABCs or ABC-like cells related to aging and autoimmune diseases. However, the existence of similarities between the roles played by these virus-induced ABC-like cells and ABCs found in aged individuals, may require further investigation.

### Altered B Cell Receptor Repertoires of the ABCs

B cell receptors (BCRs) are immunoglobulins expressed on B cell surfaces and the development of BCR repertoires is associated with the entire B cell life span (3). Primary B cell pools with great diversity are formed following development in the bone marrow. Immature B cells which leave the bone marrow continue to undergo selection based on BCR specificity. Following stimulation by antigens, mature B cells form germinal centers, in which positive selection and somatic hyper mutations occur. These B cells with high-affinity BCR will out-compete other B cells for survival signals in the germinal center (32). Class-switching can change the isotype of an antibody from IgM/IgD to IgG/IgA/IgE. Some B cells experience class-switching in the germinal centers, but such switching may also occur before the formation of germinal centers (33). These processes make the BCR repertoires more diverse and effective in their immune response. Meanwhile, B cell selections in the bone marrow and the peripheral lymphoid organs contribute to lower autoimmunity (34).

Considering that BCRs form the basis of antigen recognition by B cells, and that its sustained signaling is required for the survival of both immature and mature B cells (35), BCR repertoires are of vital importance for directing intrinsic immune responses appropriately. Thus, it may be vital to explore the properties of BCRs of ABCs. However, only a few studies have focused on this aspect. It has been shown that mutation of *VH* and *V*κ in ABCs was increased compared to that in follicular and marginal zone B cells, but at a lower frequency than in germinal center B cells of immunized mice. The mutation of BCRs in ABCs appears to be in the mid range between naïve B cells and germinal center B cells (11). Although these findings indicate altered BCR repertoires of ABCs, which may be helpful in understanding the nature of ABCs, further details regarding other characteristics of BCR repertoires of ABCs are needed.

### Functions of ABCs

**Figure 2:**
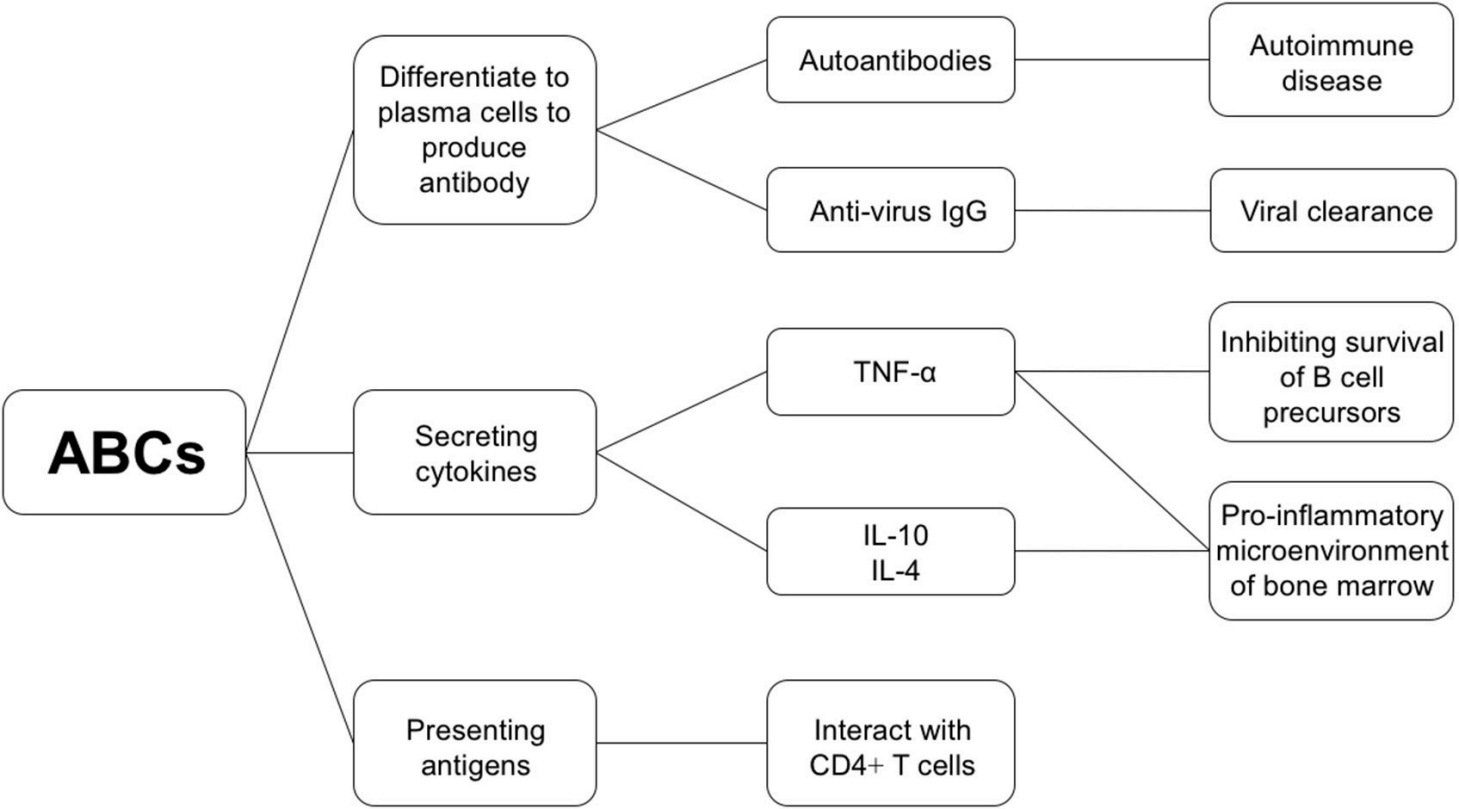
Functional properties of ABCs.

B cells are chiefly responsible for maintaining humoral defense. In addition to providing antibodies, B cells produce certain cytokines and present antigens to CD4^+^ T cells. Considering that the proportion of ABCs tends to increase with age, and that their characteristics are distinct from other B cell subsets, investigating the characteristics of their functions is important. A better understanding of their functions may help in clarifying possible mechanisms underlying immune senescence. Recent studies have demonstrated the potential function of ABCs (Figure 2).

Firstly, unlike follicular B cells, ABCs responded only to TLR7 and TLR9 stimuli *in vitro*. They were found to secrete antibodies upon TLR stimulation rather than upon BCR stimulation (9, 10). Since TLRs are commonly associated with skewing toward inflammatory responses (36), increased numbers of ABCs may yield more innate immune responses, characterized by low-affinity antibody, and inflammatory processes. Furthermore, ABCs directly participate in producing autoantibodies, indicating that they are associated with serious autoimmunity seen in the aged (10).

Secondly, the ability to secrete specific cytokines is also a critical part of ABC function. ABCs preferentially secrete IL-4 and IL-10 upon TLR stimulation *in vitro* (9), and increase the expression of TNF-α at mRNA level in mice (28). IL-4 could promote B cell maturation, which may play a role in the altered proportions of different B cell subsets (37). TNF-α was associated with a decrease in pro-B cells in the bone marrow. Co-culture of bone marrow cells with splenic ABCs from old mice showed that growth of B cell precursors was inhibited by ABCs. However, inclusion of neutralizing anti-TNF-α antibodies, prevented inhibition of B cell precursor growth by ABCs, indicating that inhibition by ABCs is mediated by TNF-α (4). Meanwhile, inhibition could also be reversed by caspase 3 inhibitor, demonstrating that TNF-α kills B cell precursors via apoptosis (4). These *in vitro* studies may provide a reasonable explanation for the reduction of bone marrow B cells, proportionately to increased ABC to follicular B cell ratio. However, relevant *in vivo* experiments are needed to validate this mechanism. Furthermore, addition of IL-10 rescued the suppression of B cell precursors induced by TNF-α (4). Detailed mechanisms underlying the effect of ABC-derived cytokines on the composition and function of B cell compartments need to be further evaluated.

Thirdly, ABCs display an enhanced ability to take up, process, and present antigens to T cells. This ability is attributed to their higher levels of MHCII and costimulatory molecules, longer and more stable interactions with T cells, more expression of genes associated with vesicular transport and cytoskeletal rearrangement and higher levels of CD11c (10, 12). In contrast to follicular B cells which are located at B cell follicles, ABCs are localized in either T cell zones or at T cell/B cell borders. This is ascribed to the finding that while ABCs convert from follicular B cells, CCR7 receptor expression in ABCs is upregulated, increasing their responsiveness to T cell zone chemokines CCL19 and CCL21, and potentiating their migration toward T cell zones (12). Such special localization causes them to be more competent in interacting with T cells. As they are capable of presenting antigens efficiently, they may function as antigen-presenting cells in the process of autoimmunity as self-antigen concentrations are usually low.

Considered together, ABCs appear to play multiple roles in age-associated alteration of immune activity. However, antigen-presentation ability is mainly displayed in *in vitro* assays. Interaction of ABCs with the other immune cells *in vivo* may need further exploration.

### Survival of ABCs Is BLyS-Independent, but IL-21 and T-Bet Are Key Factors for the Generation of ABCs

As discussed above, ABCs are likely involved in impaired immune response associated with aging. Therefore, it is important to determine their precursors, define their homeostatic requirements, and determine factors regulating their generation, as this knowledge may assist in identifying potent targets for clinical application.

In order to determine the origin of ABCs, peripheral B cell subsets in aged mice were accessed after auto-reconstitution following sublethal irradiation. If ABCs are produced by aged B lymphopoiesis, auto-reconstitution would result in their replenishment. However, compared with that of untreated aged mice their ABC level was lower, indicating that ABCs did not directly emanate from B cell genesis in the aged bone marrow (9). Studies have revealed that follicular B cells acquired the ABC phenotype only after extensive proliferation, driven by toll-like receptor (TLR) stimuli alone, or in combination with BCR stimuli, both *in vivo* and *in vitro*. This indicated that ABCs may partly originate from exhaustive expansion of mature B cells (9). *In vivo* studies using mouse models further revealed that generation of ABCs required endogenous antigen presentation via MHC class II and stimulation via the CD40 receptor (11). In summary, these studies indicate two possible sources of ABCs. One is a TLR-mediated origin, possibly through virus or autoimmune stimulation (30). The other is the accumulation of mature B cells that have undergone environmental antigen stimulation through T-dependent mechanisms.

B lymphocyte stimulator (BLyS) cytokine family, a member of the tumor necrosis factor superfamily, is critical for the survival and homeostasis of mature B cells (38). BLyS family is composed of at least two ligands, BLyS and APRIL, and three receptors, BR3, TACI, and BCMA. Among them, BLyS-BR3 signaling is crucial for naïve mature B cell survival. B cells with a different BLyS binding capacity must compete for limited survival signals (38, 39). Compared with other B cell subsets, ABCs seem not to rely on BLyS for survival, although they express BLyS receptors and are able to sequester BLyS (9). In this regard, accumulation of ABCs may reduce the availability of BLyS for other B cell subsets, gradually insulting adaptive immune response in the elderly and the homeostasis of B cells sustained by BLyS. However, more studies are required to confirm this.

In regard to factors regulating ABC generation, it has been recently reported that the transcription factor—T-bet, is highly expressed in ABCs (40). T-bet expression in B cells is essential for the production of autoantibodies (41), formation of IgG2a memory, and IgG2a class switching (42). T-bet also participates in the transition to CD11c^+^ B cells, which are major precursors for IgG2a antibody production (30). IgG2a represents the most competent isotype for antibody-dependent cellular cytotoxicity (43) and plays an important role in antiviral immune response (44). The signaling system regulating T-bet expression mainly consists of TLR stimulation, BCR stimulation, and related cytokines (45). Among these three factors, TLR stimulation is the most potent inducer, and T-bet expression is the strongest upon simultaneous stimulation by all three factors (30), suggesting that although BCR stimulation and cytokines are not indispensable, they are capable of amplifying the effect mediated by TLR stimulation.

Considering that B cell differentiation could be regulated by certain cytokines, it is interesting to know how specific cytokines affect the generation of ABCs. Evidence suggesting that cytokines play a role in the generation of ABCs come from both *in vitro* and *in vivo* studies by Naradikian et al. (40). The authors cultured follicular B cells from mice or humans with IL-4, IL-21, or IFN-γ in the presence of TLR7 or TLR9 agonists. They found that both IL-21 and IFN-γ directly promoted ABC production while IL-4 antagonized T-bet induction. These interactions of cytokines were also demonstrated *in vivo* using the influenza virus and *H. polygyrus* infections (40).

Furthermore, using transgenic mouse lupus models, it was demonstrated that interferon-regulatory factor 5 (IRF5) would control the generation of ABCs via stimulation of IL-21 (46). Considered together, both IL-21 and T-bet are key factors for ABC functioning, indicating great potential for these two factors in applications related to targeted treatment of ABC-associated diseases. Notably, T_FH_ cells are important sources of IL-21 (46). Furthermore, studies showed that co-stimulation via the CD40 receptor on B cells and CD40L on T cells may be important for ABC generation (11). Thus, the T-B interaction may be required for the generation of ABCs.

## ABCs and Autoimmune Diseases

As mentioned above, ABCs are known to secrete autoantibodies. Elevated levels of serum anti-chromatin IgG2a were observed in mice with cGVHD-induced lupus (47). *in vitro* studies also showed that ABCs preferentially induced Th17 differentiation (9). It may be important to know whether accumulation of ABCs plays a role in autoimmune diseases. Examination of mouse autoimmune-prone models (NZB/WF1 and Mer^−/−^ mice) indicated enhanced ABC populations (10, 48), raising the issue of whether ABCs lead to autoimmune diseases or emerge as byproducts of the disease process. An in-depth study demonstrated that in mouse models subjected to conditional deletion of T-bet, the formation of germinal centers was impaired, serum IgG2a levels were significantly reduced, and kidney damage as well as rapid mortality were inhibited in systemic lupus erythematosus (SLE) mice (49). Similar to the development of ABCs with aging, IL-21 may drive the generation of T-bet^+^ ABCs in autoimmune models (46). ABCs were also found in SLE patients, which was consistent with similar findings in mouse models (13). While ABCs in a fraction of these SLE patients expressed IgD, an equal percentage expressed IgG and IgA (13), indicating that some ABCs had undergone class-switching. Moreover, these cells could differentiate into plasma cells capable of producing autoantibodies (13). In addition, assessment of somatic hypermutation levels in mouse autoimmune models demonstrated that ABCs had undergone somatic hypermutation in VH, which exhibited clonal diversification of their VH genes (50)_._ However, more detailed studies are felt to be required for further substantiation.

These humans and mice studies indicate that increased numbers of ABCs may contribute to the onset and development of autoimmune diseases, which may be interpreted from two perspectives. On the one hand, TLR stimulation is a well-established factor in autoantibody production and the development of autoimmune diseases (51, 52), and ABCs have been found to produce anti-chromatin antibodies upon TLR stimulation *in vitro* (10). On the other hand, *in vitro* studies showed that ABCs preferentially skewed activated CD4^+^T cells to a Th17 fate compared to presentation mediated by young follicular B cells, aged follicular B cells or dendritic cells (9). As Th17s secrete a range of cytokines that amplify inflammatory or autoimmune diseases (53, 54), this may enable ABCs to indirectly contribute to autoimmune diseases. More researches are required to validate these hypotheses.

More interestingly, an ABC-like B cell subset, CXCR5^−^CD21^−^CD11c^+^, was described in SLE patients (14). These cells, termed double negative 2 cells (DN2 cells), are abundant in SLE patients in an age-independent pattern and capable of differentiating into autoantibody-producing plasma cells which are strongly associated with autoimmune disease (14). Similar to ABCs, DN2 cells express a T-bet transcriptional network and respond intensely to TLR7 stimuli.

Other than ABCs or ABC-like B cells, several novel subsets of B cells related to autoimmune diseases have recently been found. A novel population of memory B cells, which lack expression of CD27 and IgD, were found to be associated with disease activity and clinical manifestations of lupus in SLE patients, (15). Furthermore, CD19^hi^CXCR3^hi^ B cells in SLE are reportedly related to poor clinical outcomes following rituximab treatment (16). FcRL4^+^ B cells produce RANKL, which is associated with bone erosion in rheumatoid arthritis (17). Although associated with different markers, these B cells likely belong to the same cell type as ABCs, which needs to be confirmed by evaluating the expression of key transcription factor T-bet.

Autoimmune diseases are common diseases that threaten the health of ~23.5 million (7%) people in the United States. Women are more commonly affected than men at a significantly lower age of onset (48). Current treatment for autoimmune diseases involve immunosuppressive drugs that dampen immune responses. However, large quantities of normal cells are killed during the treatment process, resulting in lowered resistance against pathogens and higher rates of infection and cancer. Targeted therapies have also been applied to autoimmune diseases. For example, rituximab is a chimeric monoclonal antibody that targets CD20-positive B lymphocytes, leading to B cell depletion. It has been proved effective in rheumatoid arthritis and multiple sclerosis (55, 56). These treatments may also kill non-pathogenic B cells. Therefore, it may be important to find alternative targeted therapies. The association between these novel- B cells and autoimmune diseases, indicate that these cells may have great potential as therapeutic targets which may be utilized to improve treatment. Further relevant studies are felt to be needed to determine whether ABCs are the byproduct or the cause of the disease process.

## Summary

Immune cell generation as well as subset composition and function change with age. Alteration of the immune system may contribute to increased morbidity and mortality in the elderly population (57). Age-related changes in B cells are involved in this process. Accumulation of a unique B cell population that express T-bet, ABCs, is one of the most significant changes in B cell compartments. ABCs, with their inherent capacity for secreting antibodies, cytokines, and presenting antigens, may play an important role in the complicated signaling network associated with immune senescence. Moreover, ABCs appear to be associated with the onset and development of autoimmune diseases. This indicates the feasibility of ABC-targeted therapeutic approaches. Future research on the properties and functions of ABCs as well as ABC associated signaling pathways may assist us to better understand the association between ABCs and aging as well as autoimmunity. It is confirmed that TLR stimulation and related cytokines such as IL-21 are required for the expression of T-bet and generation of ABCs. However, further investigation of detailed survival requirements and functional attributes of ABCs may be required, to help design optimal clinical procedures to overcome immune senescence and autoimmune diseases in the future.

## Author Contributions

SM and YH determined the topic of the review and designed the structure of the literature. SM, CW, XM, and YH wrote the manuscript. All authors read the manuscript.

### Conflict of Interest Statement

The authors declare that the research was conducted in the absence of any commercial or financial relationships that could be construed as a potential conflict of interest.
